# Predictors of additional intraocular pressure reduction in patients changed to latanoprost/timolol fixed combination

**DOI:** 10.1186/1471-2415-10-10

**Published:** 2010-03-26

**Authors:** Eric Sellem, Jean François Rouland, Christophe Baudouin, Alain Bron, Philippe Denis, Jean-Philippe Nordmann, Jean Paul Renard

**Affiliations:** 1 Centre Ophtalmologique Kléber, 50, Cours Franklin Roosevelt, 69006 Lyon, France; 2 Ophthalmology Department, Hôpital Huriez, 1, Place de Verdun, 59037 Lille, France; 3 Ophthalmology Department 3, Centre Hospitalier National des Quinze-Vingts, 28, Rue de Charenton, Paris Cedex 12, France; 4 Ophthalmology Department, Hôpital Général, 3, Rue du Faubourg Raines, 21033 Dijon, France; 5 Ophthalmology Department, Hôpital Edouard Herriot, 5, Place d'Arsonval, 690437 Lyon Cedex 03, France; 6 Ophthalmology Department 2, Centre Hospitalier National des Quinze-Vingts, 28, Rue de Charenton, Paris Cedex 12, France; 7 Ophthalmology Department, Hôpital d'Instruction des Armées du Val de Grâce, 74, Boulevard du Port Royal, Paris Cedex 05, France

## Abstract

**Background:**

Given the growing number of ocular hypotensive medications available, it is important to be able to predict a positive response to therapy. The purpose of the present study was to identify predictors of an additional 10% intraocular pressure (IOP) reduction after 12 weeks of treatment with latanoprost/timolol fixed combination (FC) in patients requiring a change in their previous ocular hypotensive medication.

**Methods:**

This multicenter, open-label, prospective, phase IIIb study included subjects ≥18 years of age with open-angle glaucoma (OAG) or ocular hypertension (OHT). Eligible subjects had baseline IOP ≥21 mmHg and insufficient response to current beta-blocker monotherapy. The primary efficacy analysis (logistic regression) identified predictors of a positive response after 12 weeks of latanoprost/timolol FC.

**Results:**

The intent-to-treat (ITT) population included 383 subjects treated with ≥1 drop of FC and having ≥1 follow-up IOP assessment. Mean IOP was 22.19 ± 2.16 mmHg at baseline and was reduced by 5.42 ± 2.71 mmHg at study end. In all, 325 (84.9%) subjects had a positive response to latanoprost/timolol FC; the response rate was similar across groups: OAG (n = 208; 82.7%); OHT (n = 161; 87.6%); OAG+OHT (n = 14; 85.7%). Higher baseline IOP (odds ratio: 1.284; 95% confidence interval [CI]: 1.101, 1.497; p = 0.0014) and absence of adverse events (odds ratio: 0.318; 95% CI: 0.161, 0.629; p = 0.0010) were significant predictors of positive response. Age, gender, ethnic origin, diagnosis, family history of OAG/OHT, corneal thickness, and concomitant systemic beta-blocker were not significant predictors of a positive response in the ITT analysis. The FC was well tolerated. The most common adverse events were related to the eye and were consistent with known adverse events associated with latanoprost and timolol.

**Conclusions:**

These results support the use of latanoprost/timolol FC in patients whose IOP is insufficiently controlled on beta-blocker monotherapy. Patients with higher baseline IOP levels and who do not experience adverse events while on therapy are most likely to achieve a positive response to latanoprost/timolol FC.

**Trial Registration:**

Study registration number: NCT00230763

## Background

Disease progression in patients with open-angle glaucoma (OAG) or ocular hypertension (OHT) can be slowed or stopped by reducing intraocular pressure (IOP) levels though the use of ocular hypotensive agents [1-5]. First-choice options for medical treatment of elevated IOP levels usually are topical beta-blockers such as timolol maleate and prostaglandin analogs such as latanoprost [6]. Many patients require more than one IOP-lowering drug [5] resulting in complex medication regimens that may be difficult to maintain and that can lead to non-compliance [6]. Physicians should try to simplify medication regimens in order to maximize patient compliance [7-10]. In these cases, combining two medications into a fixed combination (FC) is preferable to prescribing two individual therapies [6]. Where individual treatment is available in a FC, ease of use and potential reduction of side effects argue in favor of their use [6].

Latanoprost/timolol FC became commercially available in France in 2002 and is indicated for reduction of IOP in patients with OAG and OHT who are insufficiently responsive to monotherapy with topical beta-blockers or prostaglandin analogs [11]. This FC has been shown to be effective and well tolerated [12-17]. Given the growing number of ophthalmic medications available, our goal was to identify predictors of a positive response (i.e., an additional l0% IOP reduction) in patients with OAG or OHT who required a change in their previous ophthalmic medication and who were switched to latanoprost/timolol FC.

## Methods

### Study design

This was a prospective, open-label, multicenter, phase IIIb study conducted in France between October 8, 2005, and October 5, 2007 (NCT00230763). The final protocol and informed consent documents were reviewed and approved by the Independent Ethics Committee (Comité Consultatif de Protection des Personnes dans la Recherche Biomédicale de Lyon B, France). The study complied with all International Conference on Harmonization Good Clinical Practice guidelines and with the Declaration of Helsinki. Written informed consent was obtained prior to a subject entering the study (i.e., before initiation of protocol-specified procedures).

### Subjects

Male or female subjects ≥18 years of age diagnosed with OAG or OHT with an IOP ≥21 mmHg were eligible if they were being treated with an ophthalmic beta-blocker monotherapy and if, in the investigator's opinion, they required a change in ocular hypotensive therapy because of an insufficient response to treatment. Excluded were those currently treated or treated within the prior month with any ophthalmic hypotensive agent other than a beta-blocker; with any contraindication to latanoprost or timolol including medical conditions that would preclude use of the study medication; with known intolerance to benzalkonium chloride or any excipient contained in the study medication; with severe visual field loss and/or optic disc damage; who had participated in another clinical trial within the prior 30 days; who had been previously treated with latanoprost/timolol FC; or who, in the opinion of the investigator, had an ophthalmic or general medical condition that prevented participation. Also excluded were women of childbearing potential who were not using adequate contraceptive methods or who were pregnant or nursing.

### Treatments and assessments

At the baseline visit, demographic data and medical, ocular, and treatment histories were recorded, and biomicroscopy and ophthalmoscopy were performed. Prior to pupil dilation, best-corrected visual acuity was measured (Monoyer scale for distant vision and Parinaud scale for near vision), and IOP was measured by pulse air tonometry or calibrated Goldmann applanation tonometry. With pulse air tonometry, three IOP measurements were performed, and the mean value was used in analyses; with Goldmann applanation tonometry, only one measurement was required. Both eyes were examined, even if only one eye was to be treated with latanoprost/timolol FC. The Glaucoma Symptom Scale (GSS) [18] and a satisfaction questionnaire developed for this study that included items regarding medication compliance were completed by the subject. The GSS, a brief, simple, and reliable instrument, has been found to discriminate well between individuals with and without glaucoma [18]. The GSS has been used to evaluate the personal burden of glaucoma in a variety of settings [19-21].

At the baseline visit, subjects stopped the previous ocular hypotensive therapy and were given sufficient latanoprost/timolol FC for the next 4 weeks. Subjects were instructed to instill the FC in each affected eye once daily in the evening starting the following day. Approximately 4 weeks postbaseline, subjects returned for an intermediate study visit at which time IOP was measured prior to pupil dilation using the method employed at baseline. The subject completed the GSS and satisfaction/compliance questionnaire, and physicians assessed the presence of adverse events and the global response to FC treatment. Each adverse event whether observed, elicited, or reported spontaneously was recorded, and the investigator graded its intensity (mild, moderate, severe) and evaluated whether the event was related to the FC. Serious adverse events were those that resulted in death, that were life-threatening, that required or prolonged hospitalization, that resulted in persistent or significant disability/incapacity, or that resulted in congenital anomaly/birth defect. At this intermediate visit, subjects were given sufficient latanoprost/timolol FC for the next 8 weeks. After 12 weeks of FC therapy, subjects returned for a final study visit at which time the assessments done at week 4 were repeated, visual acuity was measured, ophthalmoscopy and biomicroscopy were performed, and ophthalmic abnormalities were summarized by severity.

### Endpoints and analyses

The primary efficacy analysis was performed on the intent-to-treat (ITT) population defined as all subjects who received at least one dose of latanoprost/timolol FC and who had at least one on-study IOP evaluation. The per protocol (PP) population excluded those in the ITT population with a major protocol deviation. In analyses of IOP level, visual acuity, and GSS scores, the mean value from both eyes was used if both eyes were treated with latanoprost/timolol FC; otherwise, only data for the treated eye were included in analyses. The last observation carried forward method, using the last available postbaseline observation, was used to impute missing continuous data. The safety population included subjects who received at least one dose of latanoprost/timolol FC.

The primary efficacy endpoint was a positive response to latanoprost/timolol FC defined as an IOP reduction >10% from baseline after 12 weeks of treatment. A stepwise logistic regression model was used to evaluate associations between possible baseline and on-treatment predictors of a positive response. Potential predictors included age, gender, ethnic origin, family history of OAG, family history of OHT, concomitant systemic treatment with beta-blockers, diagnosis (OAG/OHT/OAG in one eye and OHT in the other eye), IOP at baseline, corneal thickness, compliance, and presence/absence of adverse events as well as interactions between gender and diagnosis. Subjects with missing values for predictors were excluded from analyses of affected variables. Variables with ≥20% missing data were excluded. Covariates were included in the stepwise model with the significance level for entry fixed at 5% and the significance level for retention fixed at 10%. P-values and 95% confidence intervals (CIs) for the odds ratios were calculated. In case of a difference of >10% between the number of subjects in the ITT and PP populations, a secondary analysis for the primary efficacy endpoint was conducted on the PP population.

Secondary efficacy endpoints were summarized for the ITT population overall and stratified by gender and diagnosis. Endpoints included absolute and relative IOP changes from baseline to week 12; percentages of subjects achieving a ≥5% or ≥15% IOP reduction from baseline at week 12; percentages of subjects achieving IOP levels of <18 and <16 mmHg at week 12; absolute and relative visual acuity changes from baseline to week 12; absolute changes in GSS scores (total score, non-visual symptom score, and visual symptom score) from baseline to weeks 4 and 12; subject satisfaction with FC treatment at baseline and weeks 4 and 12; and investigator's global assessment of response to FC treatment at weeks 4 and 12. The significance of potential predictors of achieving specified percentage IOP reductions and of reaching specified IOP levels at week 12 was analyzed by stepwise logistic regression models. The same 11 covariates that were included in the primary analysis were evaluated. Absolute and relative IOP changes, visual acuity changes, and changes in GSS scores were evaluated using analysis of covariance (ANCOVA) models that included the relevant baseline score as a covariate; adjusted (least square) and unadjusted means were calculated, and pairwise differences between etiologies were summarized along with standard errors, p-values, and 95% CIs. GSS scores were transformed to a scale of 0 (maximum complaint) to 100 (no complaint), where transformed score = score*25. Data concerning patient satisfaction/compliance and investigators' assessments of global response to treatment were tabulated and summarized. At baseline, the number of subjects on each type of beta-blocker was summarized as were IOP levels prior to beta-blocker initiation (if available), beta-blocker treatment duration, and reason for treatment change (investigator judgment).

Data reflecting systemic and ophthalmic adverse events were tabulated and summarized for the safety population. Adverse events were classified by System Organ Class and preferred term according to the Medical Dictionary for Regulatory Activities (MedDRA) Version 11.1. Percentages of subjects with relevant ophthalmic abnormalities at week 12 were compared across etiologies using the chi-square or Fisher's exact test.

The sample size calculation was based on the assumptions that 20% to 50% of subjects would achieve IOP reduction >10% after 12 weeks of latanoprost/timolol FC and that the same percentage of subjects was exposed to the predictive factor identified (e.g., age above n years). With an odds ratio = 2, a significance level = 5%, and power = 80%, 395 subjects were required.

## Results

**Figure 1:**
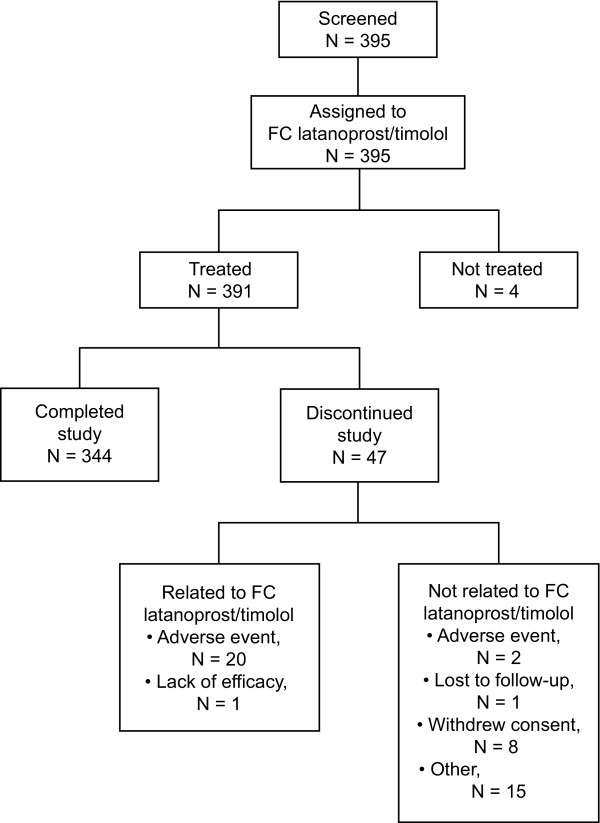
**Subject disposition**. FC = fixed combination.

One hundred and twenty-one centers screened 395 subjects and assigned all of them to latanoprost/timolol FC (Figure 1). In all, 391 subjects received at least one dose of the FC and were included in the safety population; 344 completed the study while 21 discontinued for reasons related to the study drug and 26 discontinued for reasons unrelated to latanoprost/timolol FC. The ITT population included 383 subjects, and the PP population included the 316 subjects without a major protocol deviation. Of the 98 major protocol deviations recorded in 70 subjects, 67 of whom were in the ITT population, most related to noncompliance with inclusion/exclusion criteria (n = 50), not using study drug at the time of IOP assessment (n = 20), or use of commercial latanoprost/timolol FC during the study (n = 20).

The treatment group, which was overwhelmingly Caucasian, included 182 males and 209 females with a mean age of 63.0 years (Table 1). At screening, the majority of subjects were diagnosed with OAG, more than half had at least one concomitant condition at screening, and most were being treated with timolol (206/383, 53.8%) or carteolol (144/383, 37.6%). Subjects had been on the screening therapy for an average of 2.5 years and had a mean IOP of approximately 24 mmHg in both eyes prior to initiating beta-blocker monotherapy. Insufficient efficacy was the primary reason given by investigators for switching to latanoprost/timolol FC. The median duration of latanoprost/timolol FC treatment was 89.0 days (range, 3 to 183 days).

**Table 1:** Demographic and baseline characteristics of subjects assigned to treatment, N = 391

**Gender, n (%)**	
Male	182 (46.5)
Female	209 (53.5)
**Age, years**	
Mean ± SD	63.0 ± 11.8
Range	18 - 87
**Race, n (%)**	
Caucasian	381 (97.4)
Other	10 (2.6)
Primary diagnosis*	
OAG, n	227
Time since diagnosis, years, Mean (Range)	5.0 (0.0 - 25.2)
OHT, n	181
Time since diagnosis, years, Mean (Range)	4.1 (0.0 - 24.5)
**Family history of OAG or OHT, n (%)^†^**	
Yes	208 (54.3)
No	162 (42.3)
Unknown/not recorded	13 (3.4)
**Central corneal thickness^†§^**	
Mean ± SD	559.0 ± 37.6
Range	450 - 662
**Concomitant conditions, n (%)**	
Any such condition	217 (55.5)
Vascular disorder	126 (32.2)
Ophthalmic disease	116 (29.7)
Metabolism and nutrition disorder	102 (26.1)

*Seventeen subjects had different diagnoses between eyes.

^†^Intent-to-treat population: n = 383.

^§^Data not recorded for 17 subjects.

OAG = open-angle glaucoma; OHT = ocular hypertension; SD = standard deviation.

At week 12, a positive response (IOP reduction >10% from baseline) to latanoprost/timolol FC was obtained in 325/383 (84.9%) subjects in the ITT population (Table 2). No clinically significant difference in responder rates was noted with regard to diagnosis or gender. Of the 10 potential predictors included into the logistic regression analysis, two were associated with a positive IOP response: higher baseline IOP (parameter estimate, 0.25; odds ratio [95% CI], 1.284 [1.101, 1.497]; p = 0.0014) and absence of adverse events (parameter estimate, -1.15; odds ratio [95% CI], 0.318 [0.161, 0.629]; p = 0.0010). (Note that the eleventh variable, compliance, was not included in this analysis because data were missing for ≥20% of subjects.) In the PP population, 288/316 (91.1%) subjects had a positive response. In this analysis population, higher baseline IOP but not absence of adverse events was significantly associated with a positive response to latanoprost/timolol FC; age was included in the model (p = 0.0332), but the odds ratio was close to 1 (1.045), suggesting that age had little clinical relevance.

**Table 2:** Subjects achieving a positive response to latanoprost/timolol FC* (ITT population)

	All N = 383	OAG N = 208	OHT N = 161	Both N = 14
**Positive response, n (%)**	325 (84.9)	172 (82.7)	141 (87.6)	12 (85.7)
Male, N = 179	156 (87.2)			
Female, N = 204	169 (82.8)			

*Positive response = >10% IOP reduction from baseline to week 12.

Both = both OAG and OHT; FC = fixed combination; IOP = intraocular pressure; ITT = intent-to-treat population; OAG = open-angle glaucoma; OHT = ocular hypertension.

The relative overall mean IOP reduction from baseline to week 12 of 24.10 ± 11.35% reflected a mean absolute change of -5.42 ± 2.71 mmHg from the baseline mean of 22.19 ± 2.16 mmHg (Table 3). The hypotensive effect was observed as soon as the week 4 visit when the mean IOP level was 16.98 ± 2.46 mmHg. Absolute and relative IOP changes were similar across diagnosis and gender groups. Results of ANCOVA confirmed that diagnosis did not significantly influence IOP changes.

**Table 3:** IOP changes from baseline to week 12 by diagnosis and gender, mean ± SD (ITT population)

		Diagnosis		
		
IOP	All N = 383	OAG N = 208	OHT N = 161	Both N = 14
Baseline, mmHg	22.19 ± 2.16	21.95 ± 2.27	22.40 ± 1.97	23.36 ± 2.19
Week 12, mmHg	16.77 ± 2.62	16.61 ± 2.77	17.00 ± 2.48	16.64 ± 1.67
Change from baseline to week 12, mmHg	-5.42 ± 2.71	-5.34 ± 2.76	-5.40 ± 2.60	-6.71 ± 2.95
Change from baseline to week 12, %	-24.10 ± 11.35	-24.06 ± 11.70	-23.82 ± 10.92	-28.07 ± 10.86


	**Gender**			
		
	**Male** **N = 179**		**Female** **N = 204**	
	
Baseline, mmHg	22.29 ± 2.13		22.10 ± 2.20	
Week 12, mmHg	16.53 ± 2.55		16.99 ± 2.67	
Change from baseline to week 12, mmHg	-5.76 ± 2.77		-5.11 ± 2.62	
Change from baseline to week 12, %	-25.51 ± 11.38		-22.87 ± 11.20	

Both = OAG and OHT; IOP = intraocular pressure; ITT = intent-to-treat population;

OAG = open-angle glaucoma; OHT = ocular hypertension; SD = standard deviation.

Reductions in IOP from baseline to week 12 of at least 5% or 15% were obtained in 339/383 (88.5%) and 298/383 (77.8%) of subjects, respectively (Table 4). Thus, 298 of 325 responders (91.7%) also achieved a 15% or greater decrease in IOP. Logistic regression analyses revealed that, as for the primary endpoint, two factors predicted a reduction in IOP of ≥5% and ≥15%: higher IOP at baseline and absence of adverse events (p = 0.02 for all). Again, neither diagnosis nor gender was a significant predictor of response. An IOP level <18 mmHg was achieved by 255/383 (66.6%) subjects, and an IOP <16 mmHg was noted in 128/383 (33.4%; Table 4). Lower baseline IOP and thinner corneas were significant predictors of both IOP <16 mmHg and <18 mmHg; absence of adverse events was significantly associated with reaching an IOP of <18 mmHg.

**Table 4:** Subjects achieving prespecified IOP outcomes by diagnosis and gender, n (%) (ITT population)

		Diagnosis		
		
Patients with:	All N = 383	OAG N = 208	OHT N = 161	Both N = 14
≥5% IOP reduction from baseline to week 12	339 (88.5)	182 (87.5)	145 (90.1)	12 (85.7)
≥15% IOP reduction from baseline to week 12	298 (77.8)	156 (75.0)	131 (81.4)	11 (78.6)
IOP <16 mmHg at week 12	128 (33.4)	76 (36.5)	49 (30.4)	3 (21.4)
IOP <18 mmHg at week 12	255 (66.6)	138 (66.3)	107 (66.5)	10 (71.4)


	**Gender**			
	**Male** **N = 179**		**Female** **N = 204**	
	
≥5% IOP reduction from baseline to week 12	160 (89.4)		179 (87.7)	
≥15% IOP reduction from baseline to week 12	146 (81.6)		152 (74.5)	
IOP <16 mmHg at week 12	64 (35.8)		64 (31.4)	
IOP <18 mmHg at week 12	126 (70.4)		129 (63.2)	

Both = OAG and OHT; IOP = intraocular pressure; ITT = intent-to-treat population;

OAG = open-angle glaucoma; OHT = ocular hypertension.

On average, subjects had good visual function at baseline (Monoyer scale, 8.50 ± 1.99; Parinaud scale, 2.30 ± 1.18) and experienced little change by week 12 (change in Monoyer scale, 0.03 ± 0.80; change in Parinaud scale, -0.05 ± 0.71). No clinically relevant changes in glaucoma symptoms were observed between baseline and week 12 as reflected by minimal changes in the global GSS score as well as by the non-visual and visual GSS subscale scores (Table 5).

**Table 5:** Glaucoma Symptom Scale (GSS) scores at baseline and week 12 by diagnosis and gender, mean ± SD (ITT population)

		Diagnosis		
		
	All N = 383	OAG N = 208	OHT N = 161	Both N = 14
**Global GSS score**				
Baseline	86.15 ± 15.73	84.99 ± 15.89	87.29 ± 15.54	89.41 ± 15.67
Week 12	88.49 ± 14.32	86.81 ± 15.91	90.19 ± 12.50	91.59 ± 8.50
**Non-visual GSS score**				
Baseline	84.60 ± 17.09	84.70 ± 16.14	84.34 ± 18.04	86.11 ± 20.52
Week 12	86.78 ± 15.40	84.74 ± 17.05	89.08 ± 12.95	88.10 ± 14.88
**Visual GSS score**				
Baseline	87.76 ± 18.46	85.97 ± 19.95	89.54 ± 16.81	92.79 ± 11.08
Week 12	89.76 ± 17.26	88.33 ± 19.18	91.06 ± 15.19	94.64 ± 7.70


	**Gender**			
	**Male** **N = 179**		**Female** **N = 204**	
	
**Global GSS score**				
Baseline	87.28 ± 15.24		85.19 ± 16.13	
Week 12	90.05 ± 13.96		87.08 ± 14.55	
**Non-visual GSS score**				
Baseline	86.52 ± 15.77		82.95 ± 18.02	
Week 12	88.81 ± 14.17		84.90 ± 16.27	
**Visual GSS score**				
Baseline	88.15 ± 18.29		87.43 ± 18.64	
Week 12	90.81 ± 16.40		88.83 ± 17.98	

Both = OAG and OHT; ITT = intent-to-treat population; OAG = open-angle glaucoma;

OHT = ocular hypertension; SD = standard deviation.

At the end of the study, 292/319 (91.5%) subjects responding to the satisfaction/compliance questionnaire indicated that they were "very satisfied" or "satisfied" with their ocular hypotensive therapy, an improvement from the 246/313 (78.6%) expressing this level of satisfaction with the monotherapy administered at the time of the switch to latanoprost/timolol FC. Subject-reported medication compliance (instilling drops "every day without exception") also improved from 65% under previous beta-blocker monotherapy to 81% with latanoprost/timolol FC. Nearly 90% of investigators rated the FC as "effective" at the end of the study.

No death occurred among study participants. Five subjects experienced a total of six treatment-emergent serious adverse events, none of which was considered by investigators to be treatment-related. Among the 391 subjects in the safety population, 89 (22.8%) experienced at least one all causality adverse event, and 22 (5.6%) discontinued due to an adverse event (Table 6). Eleven all causality adverse events were considered to be severe in intensity. Sixty subjects (15.3%) experienced at least one treatment-related adverse event; such events led to study discontinuation for 20 (5.1%) subjects. "Eye disorders" was the System Organ Class most frequently involved, and the most commonly reported treatment-related ocular adverse events were eye irritation (n = 5), hyperemia, (n = 4), dryness (n = 4), pruritis (n = 3), and conjunctivitis (n = 3). The incidence of ophthalmic abnormalities was similar at baseline (201/383 [52.5%]) and week 12 (195/383 [50.9%]) but was significantly greater at the end of the study among subjects with OAG versus OHT (124/195 [63.6%] versus 68/155 [43.9%], respectively; p < 0.001).

**Table 6:** Adverse events by body system, n (%)

	All causality adverse events N = 391	Treatment-related adverse events N = 391
Any adverse event	89 (22.8)	60 (15.3)
Discontinued due to adverse event	22 (5.6)	20 (5.1)

**System Organ Class**		
Cardiac disorders	3 (0.8)	2 (0.5)
Ear and labyrinth disorders	4 (1.0)	3 (0.8)
Eye disorders	40 (10.2)	34 (8.7)
Gastrointestinal disorders	6 (1.5)	3 (0.8)
General disorders and administration site conditions	11 (2.8)	8 (2.0)
Immune system disorders	2 (0.5)	2 (0.5)
Infections and infestations	8 (2.0)	1 (0.3)
Injury, poisoning, and procedural complications	2 (0.5)	1 (0.3)
Investigations	2 (0.5)	2 (0.5)
Metabolism and nutrition disorders	1 (0.3)	1 (0.3)
Musculoskeletal and connective tissue disorders	2 (0.5)	-
Nervous system disorders	12 (3.1)	8 (2.0)
Psychiatric disorders	8 (2.0)	6 (1.5)
Reproductive system and breast disorders	1 (0.3)	1 (0.3)
Respiratory, thoracic, and mediastinal disorders	4 (1.0)	3 (0.8)
Skin and subcutaneous tissue disorders	10 (2.6)	9 (2.3)
Surgical and medical procedures	2 (0.5)	-
Vascular disorders	6 (1.5)	2 (0.5)

## Discussion

The goal of this 12-week, prospective, open-label, multicenter study was to identify predictors of a positive response, defined as an additional l0% IOP reduction, to latanoprost/timolol FC in patients with OAG or OHT whose IOP was insufficiently controlled on beta-blocker monotherapy and who required a change in their previous ophthalmic medication. A positive response was observed in 84.9% subjects in the ITT population, and patients most likely to achieve such a response were those with higher baseline IOP levels and those who did not experience adverse events while on treatment. Other factors tested, including age, gender, ethnic origin, diagnosis, family history, corneal thickness, and concomitant use of a systemic beta-blocker, were not significant predictors of a positive response. However, in the PP population (which included only patients without a major protocol violation), age, but not absence of adverse events, was a significant predictor of positive response. The predictive power of initial IOP, absence of adverse events, and age with regard to IOP reduction parallels results of previous studies. Higher baseline IOP levels have been associated with greater IOP reductions, due in part to regression to the mean [22-26]. Negative associations have been found between medication-related adverse events and both compliance [7,27] and persistence [28]; it is intuitive that patients who do not take their ocular hypotensive medication as prescribed cannot obtain their full IOP-lowering benefit. Finally, older age has been identified as a risk factor for progression of glaucoma to blindness [4,29-31].

The effectiveness of latanoprost/timolol FC in reducing IOP levels has been demonstrated in randomized, double-masked, controlled clinical trials [12-17], and switch studies such as ours are not appropriate for comparing therapies with regard to efficacy. Compared with other studies with similar designs, it is notable that the mean IOP reduction of 5.4 mmHg over 12 weeks was greater than reductions reported previously [32,33]. Thus, an average IOP reduction of 3.7 mmHg (from 21.6 to 17.9 mmHg) in 2 months was reported for 53 patients switched from timolol monotherapy to latanoprost/timolol FC [32]. In a study that monitored changes in IOP during the first 6 months following a switch to the FC, Dunker et al. [33] found statistically significant mean IOP reductions of 3.3 mmHg among 902 OAG patients and 3.4 mmHg in 42 patients with OHT.

An unknown proportion of IOP reductions reported in switch studies may have been attributable to the Hawthorne effect, the tendency for subjects to improve in response to the fact of being studied rather than in response to the experimental change [34,35]. Findings of six studies [36-41] in which one group or eye continued on the baseline ocular hypotensive therapy while the other group or eye switched to another agent for periods ranging from 21 days to 6 months are inconsistent with regard to the potential magnitude of such an effect. Effect sizes ranged from -0.37 mmHg in 115 patients continued for 6 months on dual therapy that included a beta-blocker [40] to -3.1 mmHg in five patients continued for 12 weeks on timolol [38]. Mean IOP reductions in the control group in each of these six studies were substantially smaller than the -5.4 mmHg mean change from baseline to week 12 observed herein.

No change in visual acuity was noted at the end of this relatively short-term study in these patients with good vision at baseline, and no clinically relevant changes in glaucoma symptoms based on GSS scores were found. The latter result may reflect the fact that the study population presented at baseline with GSS scores similar to those observed in the reference group (without glaucoma) of the study that assessed the validity and reliability of the GSS [18]. In addition, the GSS may not be sensitive enough to assess short-term changes in symptoms in our patient group. The authors noted that the population used to test the validity and reliability of the GSS "... may not accurately represent a community-based setting, where populations of persons with glaucoma are likely to have milder disease and/or are likely to receive treatments" [18].

As has been reported previously [12-17], the FC was well tolerated. The most common adverse events were related to the eye and were consistent with known adverse events associated with latanoprost or timolol. The six serious adverse events were not considered to be related to study treatment.

While this study's open-label observational design may better reflect actual clinical practice than controlled clinical trials, the study had several limitations. In particular, not all of the investigators used applanation tonometry to measure IOP levels, and because the method of IOP measurement was not always documented, we could not analyze IOP changes stratified by tonometry type. Although every effort was made to ensure consistency of procedures and data recording across centers, the extent of variation among practices is unknown. The 12-week follow-up period was not sufficient to detect long-term changes in visual acuity or GSS scores. Finally, we considered a favorable response to the FC to be an additional 10% IOP reduction, an arbitrary cutoff point. Regression to the mean [26] may have accounted for reductions in some of the 84.9% of subjects counted as responders, but it is notable that 77.8% of subjects recorded IOP reductions of at least 15% from baseline to week 12.

## Conclusions

Reducing IOP levels slows down disease progression in glaucoma patients [1-4] and delays progression to glaucoma in OHT patients [5]. Our findings support the use of latanoprost/timolol FC in routine clinical practice in patients whose IOP is insufficiently controlled on beta-blocker monotherapy. Patients with higher baseline IOP levels and who do not experience adverse events are most likely to achieve a positive response to the FC.

## Competing interests

The authors have no proprietary interests. The study was supported by Pfizer France.

## Authors' contributions

ES participated in the study design, acquisition of data, analysis and interpretation of data, drafting of the manuscript, critical revision of the manuscript for important intellectual content, and study supervision. JFR participated in the study design, acquisition of data, and critical revision of the manuscript. CB and J-PN participated in the study design and critical revision of the manuscript. PD participated in the study design, analysis and interpretation of data, drafting of the manuscript, and revision of the manuscript. AB participated in the acquisition of data and critical revision of the manuscript. JPR participated in the study design, critical revision of the manuscript, and study supervision. All authors read and approved the final manuscript.

## Pre-publication history

The pre-publication history for this paper can be accessed here:

http://www.biomedcentral.com/1471-2415/10/10/prepub
